# BDNF Unveiled: Exploring Its Role in Major Depression Disorder Serotonergic Imbalance and Associated Stress Conditions

**DOI:** 10.3390/pharmaceutics15082081

**Published:** 2023-08-03

**Authors:** Ana Salomé Correia, Armando Cardoso, Nuno Vale

**Affiliations:** 1 OncoPharma Research Group, Center for Health Technology and Services Research (CINTESIS), Rua Doutor Plácido da Costa, 4200-450 Porto, Portugal; anncorr07@gmail.com; 2 CINTESIS@RISE, Faculty of Medicine, University of Porto, Alameda Professor Hernâni Monteiro, 4200-319 Porto, Portugal; cardosoa@med.up.pt; 3 Institute of Biomedical Sciences Abel Salazar (ICBAS), University of Porto, Rua de Jorge Viterbo Ferreira, 228, 4050-313 Porto, Portugal; 4 NeuroGen Research Group, Center for Health Technology and Services Research (CINTESIS), Rua Doutor Plácido da Costa, 4200-450 Porto, Portugal; 5 Unit of Anatomy, Department of Biomedicine, Faculty of Medicine, University of Porto, Alameda Professor Hernâni Monteiro, 4200-319 Porto, Portugal; 6 Department of Community Medicine, Information and Health Decision Sciences (MEDCIDS), Faculty of Medicine, University of Porto, Alameda Professor Hernâni Monteiro, 4200-319 Porto, Portugal

**Keywords:** major depressive disorder, brain-derived neurotrophic factor, serotonin, hypothalamic–pituitary–adrenal axis, oxidative stress

## Abstract

Brain-derived neurotrophic factor (BDNF) is a neurotrophin that plays a significant role in the survival and development of neurons, being involved in several diseases such as Alzheimer’s disease and major depression disorder. The association between BDNF and major depressive disorder is the subject of extensive research. Indeed, numerous studies indicate that decreased levels of BDNF are linked to an increased occurrence of depressive symptoms, neuronal loss, and cortical atrophy. Moreover, it has been observed that antidepressive therapy can help restore BDNF levels. In this review, we will focus on the role of BDNF in major depression disorder serotonergic imbalance and associated stress conditions, particularly hypothalamic–pituitary–adrenal (HPA) axis dysregulation and oxidative stress. All of these features are highly connected to BDNF signaling pathways in the context of this disease, and exploring this topic will aim to advance our understanding of the disorder, improve diagnostic and treatment approaches, and potentially identify new therapeutic targets to alleviate the heavy burden of depression on society.

## 1. Introduction

Brain-derived neurotrophic factor (BDNF) is a widely studied neurotrophin that is very important in several physiological processes, such as neuronal development, synaptic plasticity, neurogenesis, neuroprotection, learning, memory, and mood regulation, mainly by interacting with tropomyosin receptor kinase B (TrkB) [1,2]. Being a player in several diseases, the connection of the BDNF signaling pathway with major depressive disorder is widely studied, being very important in advancing the understanding and treatment of this disorder [3]. Nevertheless, there is still a lot to undercover in this active research field.

In the world, an estimated five percent of adults suffer from depression, a highly debilitating disease that can even lead to death by suicide [4]. Despite the existence of effective treatments, relapse after treatment is frequent, as well as resistance to several treatment options. Indeed, this is an extremely heterogeneous disorder, and around 30% of those suffering from it are resistant to standard treatments, emphasizing the importance of continuous research [5].

This disease is a complex condition characterized by numerous molecular and cellular features. While serotonin has long been associated with depression, it is important to acknowledge that there are various other factors at play, such as neuroinflammation and dysregulation in diverse neurotransmitters, such as gamma-aminobutyric acid (GABA) and glutamate [6]. Moreover, several studies have also found that reduced levels of BDNF are associated with an increased frequency of depressive symptoms. Indeed, it is known that BDNF levels can be restored with antidepressant therapy. Thus, BDNF appears to play an important role in the underlying mechanisms of depression, according to several studies [7]. Other significant mechanisms have gained recognition in understanding the process of depression, such as oxidative stress and imbalance in the hypothalamic–pituitary–adrenal (HPA) axis, important in the regulation of the stress response. In fact, studies have shown that increased oxidative stress can contribute to the development and progression of depression, as well as HPA axis dysregulation [8,9]. The interplay of oxidative stress, HPA axis imbalance, and serotonergic pathways with BDNF is also an important topic of research, playing a significant role in the development and progression of depression and other neuropsychiatric disorders [10,11,12,13]. Several studies connect these features, as described below. Thus, our review focuses on this relationship, highlighting recent studies in this field. This knowledge holds the potential to drive advancements in the diagnosis, treatment, and management of major depressive disorder, ultimately improving the lives of people who suffer from this condition.

## 2. Overview of Brain-Derived Neurotrophic Factor

BDNF, a member of the neurotrophin family, plays a vital role in the survival and differentiation of neurons during development [14]. It is synthesized primarily by neurons as proBDNF, a pre-protein weighing around 32 kDa [15]. Initially, the precursor protein, preproBDNF, is produced in the endoplasmic reticulum. Upon cleavage of the signal peptide, proBDNF (~32 kDa) is formed. Later, proBDNF is converted into its mature form, a 13 kDa polypeptide. Additionally, a BDNF pro-peptide (~17 kDa), representing the *N*-terminal fragment of proBDNF, is also produced during this process [16].

The expression of BDNF is regulated through different means at the transcriptional level. In humans, this gene has nine promoters, encoding the identical protein but producing different noncoding exons. Indeed, the human BDNF gene possesses 11 exons (I–IX, Vh, and VIIIh), and the expression of BDNF transcripts has cell and activity specificity, and distinct transcripts have distinct roles in both molecular and behavioral expression [17,18]. Exon IV is the most studied, being important for the modulation of mood, cognition, and behavior [19]. Indeed, the methylation of the promoter of this exon is a potential biomarker for antidepressant therapy in major depressive disorder [20]. Additionally, evidence indicates that miR-182 is a potential regulatory microRNA for BDNF, suggesting that serum BDNF and related miRNAs could serve as valuable biomarkers for diagnosing depression or as potential therapeutic targets [21].

BDNF plays a vital role in regulating various cellular responses by influencing synapses, promoting the growth of new neurons, facilitating the growth of axons, and ensuring the survival of neurons [16]. However, it is important to note that pro-BDNF can trigger apoptosis, decrease the density of dendritic spines, and facilitate long-term depression (LTD) in the hippocampus [1,22]. Indeed, the expression of BDNF has been observed in both the central nervous system (CNS) and the peripheral nervous system (PNS), being synthesized by neurons, oligodendrocytes, and other cells such as platelets, and T and B lymphocytes [1]. In the human and rodent brain, BDNF is widely and highly expressed in regions such as the hippocampus and cerebral cortex. Indeed, hippocampal neurons exhibit the highest levels of BDNF. Lower levels of BDNF have been detected in organs such as the liver and lung [14].

This neurotrophin exerts its effects by interacting with two types of receptors. One belongs to the family of receptors with tyrosine kinase activity, specifically TrkB, and the other is the p75 neurotrophin receptor (p75 NTR), which is a neurotrophin receptor with low binding affinity for mature BDNF [1]. When BDNF binds to the TrkB receptor, it primarily activates three intracellular signaling pathways: ERK, PI3K/Akt, and phospholipase Cγ (PLCγ) signaling. These signaling pathways are crucial for mediating the diverse functions of BDNF [23].

Research conducted on humans indicates that variations of levels of BDNF in the peripheral bloodstream are positively linked to hippocampus size and cognition, having a pivotal role in diseases such as Alzheimer’s disease, Parkinson’s disease, and Huntington’s disease [24,25]. Conversely, BDNF is also inversely associated with mood disorders, such as the above-mentioned major depressive disorder, which will be further discussed in this review. Increasingly studied, various forms of physical exercise stimulate the production of this neurotrophin, leading to cognitive enhancement and the reduction of symptoms related to depression and anxiety [26]. Indeed, interval training has been demonstrated to result in an increase in BDNF concentrations in the serum and plasma of a healthy young population [27]. Exploring BDNF’s functions and its relationship with other factors is important for advancing our knowledge of brain health and developing potential therapeutic strategies for related conditions.

## 3. The Multifaceted Roles of Brain-Derived Neurotrophic Factor

BDNF is known to play an important role in several physiological processes such as neuronal survival and differentiation, synaptic plasticity, neurogenesis, and neuroprotection. Additionally, this neurotrophin is important in learning, memory, mood regulation, and other cognitive processes. Indeed, reduced levels of BDNF contribute to cerebral atrophy, cognitive decline, and the increased risk of psychiatric disorders [2]. The release and activity of various neurotransmitters, including glutamate, GABA, and dopamine, are also influenced by BDNF, which can modulate the balance and function of neurotransmitter systems, affecting neuronal communication and overall brain function [28]. BDNF also participates in the formation of appropriate synaptic connections in the brain, being important in developing and maturing the nervous system. Indeed, this neurotrophin participates in processes such as the development of dendrites and synaptic specializations, maturation and refinement of dendritic arbors, and axon growth and differentiation [29]. Table 1 represents a summary of the main known BDNF functions.

One of the most studied and characterized roles of BDNF is the regulation of postsynaptic and presynaptic transmission, regulating synaptic plasticity [30]. This process is the capability of modifying the strength or efficacy of synaptic transmission at preexisting synapses [31]. Multiple research studies have confirmed that BDNF assumes a crucial function in hippocampal long-term potentiation (LTP), which is a sustained improvement in synaptic effectiveness believed to be the foundation of learning and memory [30]. Indeed, it is known that modifications in synaptic connections contribute to the retention of memories. Studies demonstrate that compromised BDNF function is associated with memory impairment, being associated with dementia [32]. BDNF is, thus, important for enhancing synaptic efficacy, by regulating the trafficking, phosphorylation, and expression levels of the *N*-methyl-D-aspartate receptor (NMDAR), a type of G protein-coupled ionotropic glutamate receptor that is a key player on synaptic plasticity [33]. Additionally, BDNF also influences dendritic spine characteristics and promotes neurogenesis through effects on cell survival and proliferation [14,34].

Several pieces of evidence demonstrate that BDNF plays an important role in adult neurogenesis [35], which is the creation of fully functional neurons from neural precursors in adult organisms in specific areas of the mammalian brain, particularly in the hippocampus [36]. Research has demonstrated that BDNF can enhance the multiplication of neural progenitor cells (NPCs) and facilitate the prolonged viability of their progeny [37]. BDNF administration to the dentate gyrus of adult rats led to the increased neurogenesis of granule cells [38]. Another example that highlights the connection between BDNF and neurogenesis is the treatment with *Panax notoginseng* saponins, used in the context of cerebral ischemia injury, that stimulates hippocampal neurogenesis by inducing different pathways, such as the upregulation of BDNF [39].

BDNF also presents various protective effects on the brain, such as preventing cell death, reducing oxidative damage (anti-oxidation), and inhibiting autophagy [40]. Indeed, based on the functions of this neurotrophin, there are several treatments and strategies that have been shown to increase the levels of BDNF, such as regular physical exercise, some antidepressant medications (namely serotonin reuptake inhibitors (SSRIs)), and omega-3 fatty acid intake [14,41]. Thus, BDNF is a protein that plays a crucial role in promoting the growth, development, and maintenance of neurons in the brain, being involved in various processes such as neuroplasticity, synaptic modulation, and neuronal survival. Being implicated in the pathophysiology of disorders such as Alzheimer’s disease, Parkinson’s disease, and major depression disorder, BDNF is an active area of investigation in neuroscience and related fields.

**Table 1:** Summary of BDNF main functions in the central nervous system.

Function	Description
Neuronal Development	Promotes the growth and development of neurons during early brain development, contributing to the formation of neuronal connections and neural circuits [29].
Synaptic Plasticity	Regulates synaptic plasticity. It facilitates the strengthening and formation of new synapses [30].
Learning, Memory, and Mood Regulation	Supports the formation of long-term memories and promotes the consolidation of newly acquired information. BDNF is also implicated in mood regulation, being associated with the pathophysiology of psychiatric disorders, such as major depression disorder [2,32].
Neurogenesis	Promotes the generation of new neurons, replenishing and maintaining a healthy population of neurons [35].
Neuroprotection	Helps to mitigate damage caused by oxidative stress, inflammation, and other harmful processes in the brain [40].

## 4. Exploring BDNF and Its Connection to Mood Disorders

BDNF is recognized for its vital involvement in numerous cognitive processes and has been linked to several psychiatric and neurological conditions, including major depression disorder, anxiety disorders, schizophrenia, and neurodegenerative diseases [3].

Focusing on major depressive disorder, this disease is one of the most common psychiatric disorders, with a high economic burden [42]. This is an illness characterized by symptoms such as anhedonia, sadness, disrupted sleep patterns, and cognitive abnormalities. In severe cases, it can even lead to suicide. While the molecular aspects of major depressive disorder are not fully understood, they are believed to involve deficiencies in neurotransmission, reduced levels of BDNF, genetic factors, the immune system, and hormonal imbalances, as well as environmental factors [43,44].

The relationship between BDNF and major depressive disorder is an extensive area of study with some inconsistencies between results and a lot to uncover. Nevertheless, the neurotrophic hypothesis of depression relies heavily on the connection between reduced levels of BDNF and an increased occurrence of depression and associated features [45]. Indeed, several studies state that lower levels of BDNF are correlated with a higher frequency of depressive symptoms, loss of neurons, and cortical atrophy [3]. Also, BDNF levels can be restored with antidepressive therapy [7]. Studies using animals to model depression indicate that BDNF plays a crucial role in the underlying mechanisms of this illness. In these experiments, chronic stress and depression led to a reduction in BDNF levels, an increase in cell death, a decrease in the growth of new neurons in the hippocampus, and a decrease in BDNF expression in other regions of the brain [46,47]. Additionally, several studies have found lower serum BDNF levels in patients with depression, compared to non-depressed individuals [48].

A recent study revealed that, after 100 days of selective serotonin reuptake inhibitor treatment, methylation of promoter CpG sites of BDNF was significantly decreased, reducing depression scores after this treatment [49]. Val66Met is a genetic variation that happens naturally in the BDNF gene, leading to a substitution of valine (Val) with methionine (Met) at position 66 [50]. Indeed, a recent study correlated this polymorphism with major depressive disorder, having the potential to be used as a biomarker for the prediction of response to antidepressants and electroconvulsive therapy in depressive patients. In fact, the presence of the Met allele could potentially serve as an indicator for predicting the likelihood of developing major depressive disorder [51]. Another study with behavioral tests demonstrated antidepressant effects as well as an elevation in serum BDNF levels following the prolonged use of allopurinol, recently correlated with its effects on serotonin and depression-like behaviors [52]. A further recent study also revealed that the modulation of depressive-like behaviors in chronically restrained mice is achieved through the upregulation of BDNF/TrkB signaling by the δ opioid receptor agonist SNC80 in the hippocampus and amygdala, exerting anti-depressant effects [53]. Another compound, Luteolin-7-O-Glucuronide, also significantly improved depression-like behavior by activation of BDNF signaling pathways in mice [54]. Interestingly, fecal microbiota transplantation for the treatment of depression in rats exerted an antidepressant effect by increasing the expression levels of BDNF and other components such as serotonin [55].

TrkB phosphorylation and expression also changes in depressed individuals. Indeed, antidepressants enhance TrkB signaling in the cerebral cortex, and this process relies on BDNF to manifest the behavioral benefits commonly associated with these drugs. Additionally, the tyrosines in the TrkB autophosphorylation site are phosphorylated in response to antidepressants [56]. In fact, the activated phosphorylated forms of TrkB have been found to be decreased in brain samples from depressed patients [57]. Several antidepressants, including SSRIs and ketamine, directly bind to this receptor, activating/enhancing the TrkB-BDNF signaling pathway [58]. Table 2 represents a summary of the BDNF and major depressive disorder connection.

### 4.1. Oxidative Stress and BDNF: Exploring the Role in Major Depression Disorder

Oxidative stress occurs when there is an imbalance between the production of harmful free radicals, particularly reactive oxygen species (ROS), and the body’s ability to counteract their effects. Maintaining a proper balance is crucial for the body to effectively neutralize these reactive species. Various illnesses, including cancer, diabetes, and cardiovascular and neurological disorders, arise due to the disruption of this oxidative equilibrium [11]. Indeed, the role of oxidative stress in depression is well-known, being involved in the pathogenesis of this disease and increased in individuals with depression [8,60]. The presence of oxidative stress indicators is a common observation in depressed humans and animal models of depression [61]. Also, oxidative stress is usually accompanied by excitotoxicity, a process of cell death resulting from the toxicity of excitatory amino acids, which is caused by glutamatergic NMDA receptor hyperactivation. Indeed, defective glutamate clearance and increased glutamate release by activated glial cells raise glutamate levels and disrupt signaling via glutamate receptors, contributing to neuronal dysfunction and, eventually, behavioral abnormalities observed in depression [62,63]. A meta-analysis of randomized clinical trials concluded that antioxidant supplementation is linked to reduced levels of anxiety and improved depressive symptoms [64].

Oxidative stress can lower BDNF production and damage its signaling pathways, which can harm BDNF levels. On the other hand, low levels of BDNF may exacerbate oxidative stress by impairing the brain’s antioxidant defenses, influencing each other in the context of depression and other neuropsychiatric diseases such as bipolar disorder [11,65]. BDNF is also known to increase the expression of antioxidant enzymes, particularly superoxide dismutase and glutathione peroxidase. These enzymes help counteract oxidative stress by neutralizing ROS and decreasing oxidative damage [65]. The interplay between BDNF and oxidative stress in depression is sustained by several studies. Also, several antidepressant drugs are known to elevate BDNF levels and, at the same time, reduce oxidative stress parameters. This is the case of, for example, mirtazapine [66,67,68] and escitalopram [69,70]. Another piece of evidence that highlights this connection is the increased susceptibility to stress-induced oxidative stress in the cerebral cortex of BDNF deficient mice [71]. Another study revealed that *Tagetes minuta* flower essential oil reduced oxidative stress and restored BDNF-Akt/ERK2 signaling, reducing stress- and inflammation-induced depressive-like behavior in mice [72]. In PC-12 cells, the exposition to TPPU (1-(1-propanoylpiperidin-4-yl)-3-[4-(trifluoromethoxy)phenyl]urea) reduced oxidative stress injury induced by hydrogen peroxide and promoted BDNF expression [73]. In rats, it was also demonstrated that omega-3 fatty acids could restore the balance of oxidative stress, the inflammatory response, the production of BDNF, and the metabolism of serotonin to prevent the nicotine withdrawal-induced escalation of anxiety and depression [74]. Another recent study explored the role of the antioxidant hesperetin in a reserpine-induced depression model in male rats, revealing that this compound, found in citrus peels, could significantly improve BDNF levels in the hippocampus of these animals and decrease oxidative stress levels by increasing antioxidative markers, particularly superoxide dismutase and glutathione peroxidase [75]. The same response was observed with the administration of red raspberry extract to rats by using a chronic unpredictable mild stress-induced depression model. Indeed, this extract has shown potential efficacy in reducing depressive-like behavior and histological damage to hippocampus tissue in these rats via controlling neuroinflammation, the oxidative stress response, and BDNF/TrkB levels, regulating GSK3β and mTOR signaling pathways [76].

In sum, the intricate relationship between oxidative stress and BDNF levels plays a significant role in the development and progression of depression and other neuropsychiatric disorders. Indeed, antioxidant supplementation has been linked to improved depressive symptoms. Additionally, certain natural compounds and antidepressant drugs, such as mirtazapine and escitalopram, elevate BDNF levels while reducing oxidative stress markers. These findings highlight the importance of targeting both oxidative stress and BDNF pathways as a comprehensive approach to managing depression and related neuropsychiatric disorders.

### 4.2. Exploring the Link between BDNF and HPA Axis Dysregulation: Implications for Major Depression Disorder

The HPA axis is the primary contributor to the stress response, being implicated in the pathophysiology of several mood and cognitive disorders. Indeed, major depression is associated with an overactive HPA axis, according to neuroendocrine studies [9]. High levels of glucocorticoids, such as cortisol, can have detrimental effects on several brain areas, such as the hippocampus, prefrontal cortex, and amygdala, which are involved in mood regulation [77,78]. Prolonged exposure to elevated glucocorticoid levels can lead to structural changes in these brain regions, including reduced volume and impaired functioning. These changes are thought to contribute to the development of depressive symptoms [11,79]. This axis is known to be reportedly downregulated by several antidepressants, attenuating depressive-like symptoms [80]. Synapse loss, neuronal death, and modifications to the dendrites of neurons are additional effects of prolonged exposure to high levels of glucocorticoids, produced by the adrenal gland [81].

Studies have shown interactions between HPA-axis activity and BDNF. Indeed, stress-induced HPA axis hyperactivity and the resulting increase in glucocorticoid levels diminishes BDNF expression. Also, the glucocorticoid receptor directly impacts the function of TrkB [10]. Additionally, BDNF has been found to regulate the activity of this axis, reducing its activity, potentially leading to lower glucocorticoid levels [82]. It is important to note that acute or chronic stress-induced HPA axis activation affects different BDNF signaling pathways. Indeed, it is known that with short-term stress, the temporary activation of the HPA axis can have positive effects on neurotransmission and synaptic plasticity in the prefrontal cortex. These effects are believed to improve the processes related to emotional memory and enhance the ability to cope with future stressors. On the other hand, chronic stress has detrimental effects on neuroplasticity in the prefrontal cortex and disrupts the normal regulation of the HPA axis. These negative effects increase the risk of developing mental health disorders, such as major depressive disorder [83]. BDNF gene polymorphisms are also linked to HPA axis modulation in major depressive disorder, affecting antidepressant treatment response [84].

Several recent studies highlight the connection between this axis and BDNF. In a study in mice, chronic corticosterone administration induced depressive-like behaviors and decreased the expression of BDNF in the dentate gyrus of the hippocampus [85]. In female mice, oxytocin administration reduces dexamethasone-induced depression-like symptoms through increasing hippocampus cAMP-response element binding protein (CREB)-BDNF signaling [86]. Another recent study in HT-22 cells (mice hippocampal cells) found that the total alkaloids of *Fibraurea recisa* protected these cells from corticosterone-induced damage and increased cell viability, significantly increasing the levels of BDNF [87]. Kolaviron, a biflavonoid, also protected mice from chronic unpredictable mild stress-induced anxiety and sadness by enhancing antioxidant defense mechanisms, reducing BDNF levels in the prefrontal cortex and hippocampus and decreasing corticosterone levels, which were increased after the stress exposure [88]. Another recent study in mice found that corticosterone treatment caused distinct alterations in proBDNF and mature BDNF in different brain areas. Both proBDNF and mature BDNF levels were found to be considerably higher in the pituitary gland. ProBDNF, on the other hand, was dramatically reduced in the adrenal gland. Indeed, corticosterone produced depressive behavior in mice but affected proBDNF processing differently [89].

In summary, the HPA axis plays a significant role in the stress response and is involved in the development of mood disorders. There is a complex interaction between the HPA axis and BDNF. Indeed, in a general way, chronic stress-induced HPA axis hyperactivity and elevated glucocorticoid levels can decrease BDNF expression, while BDNF can modulate HPA axis activity, controlling glucocorticoid levels. Recent studies have highlighted the association between the HPA axis and BDNF, highlighting the impact of corticosterone on BDNF levels and behavior in animal models, being extremely important in the context of major depressive disorder comprehension and treatment.

### 4.3. The Intersection of BDNF and Serotonergic Systems in Major Depression Disorder

Serotonin and serotonin receptors are involved in the control of nearly all brain activities, and serotonergic dysregulation has been linked to the pathophysiology of many psychiatric and neurological illnesses. Outside of the central nervous system, serotonin has crucial roles in many human organ systems, including the regulation of gastrointestinal, endocrine function, cardiovascular and pulmonary physiology [90]. The connection between this neurotransmitter and major depressive disorder has been widely studied for decades. Indeed, low levels of this neurotransmitter and the occurrence of this disorder are associated, mainly supported because SSRIs are effective drugs for major depressive disorder treatment [91]. Nevertheless, research suggests that a deficiency or imbalance in serotonin levels may contribute to the development of depression, despite the contribution of several other factors and some controversy in the literature [92].

Research has shown that BDNF and serotonin are interconnected in several ways. Indeed, serotonergic pathways influence the expression and release of BDNF in the central nervous system, and BDNF is involved in the regulation of the development and function of serotonergic neurons [12,13]. Also, imbalances in serotonin levels can impact BDNF levels in the central nervous system [93] and BDNF promotes serotonergic neuron development, maintenance, and plasticity, influencing serotonin synthesis and availability [12,93]. Additionally, both serotonin and BDNF contribute to the modulation of synaptic connections and structural changes in the brain during neuroplasticity, both being important in the context of mood disorders such as depression [94]. In fact, the administration of SSRIs is known to enhance BDNF gene expression [94]. A meta-analysis of the comparative efficacy of antidepressants on peripheral BDNF concentrations in patients with depressive disorder revealed that both SSRIs and serotonin and norepinephrine reuptake inhibitors (SNRIs) boosted BDNF levels after a period of treatment, and sertraline outperformed the other three medications (venlafaxine, paroxetine, or escitalopram) in terms of early BDNF concentration rise [70]. It was also demonstrated that both serotonin levels and BDNF levels are altered by early-life selective serotonin reuptake inhibitor exposure in rodents, affecting the maturation of prefrontal cortex and amygdala circuits [95]. Serotonergic receptors such as 5-HT2A have also been shown to modulate BDNF expression in limbic neurocircuits such as the prefrontal cortex and hippocampus, important in the context of major depressive disorder. Furthermore, changes in BDNF have a direct impact on 5-HT2A receptor production, signaling, and function [96]. Additionally, this receptor and its interplay with TrkB contributes to neuroplasticity regulation, implicated in numerous neuronal disorders [97]. Several psychedelics, agonists of the 5-HT2A receptor, promote plasticity by directly binding to TrkB. Indeed, these drugs exhibit rapid and long-lasting antidepressant effects while promoting neuroplasticity that bears similarities to the impact of conventional antidepressant treatments. For example, it was demonstrated that lysergic acid diethylamide (LSD) and psilocin directly bind to TrkB with affinities 1000-fold higher than fluoxetine and ketamine [98].

The fast and prolonged antidepressant-like effects of the activation of the serotonin receptor 5-HT1A require medial prefrontal cortex α-amino-3-hydroxy-5-methyl-4-isoxazolepropionic acid (AMPA) receptor and BDNF signaling [99]. In addition, a recent study revealed that BDNF and the 5-HT7 receptor also interconnect. Indeed, when activated, these receptors increase the level of BDNF and TrkB affinity [100]. Interestingly, a study in rodents demonstrated that a single injection of BDNF improved the activity of serotonin transporter in the hippocampus of these animals. Such acute BDNF effects would be predicted to counteract the early effects of SSRIs, which could explain some of the delay in their therapeutic effects [101]. Thus, understanding the intricate relationship between serotonin and BDNF is important for advancing our knowledge of major depression disorder. Recent studies on this topic explore this connection in depressive disorder. For example, after a prolonged treatment with SSRIs, the methylation of promoter CpG sites of BDNF was considerably reduced, improving treatment and reducing depression scores after treatment [49]. Another study based on reserpine-induced depressive-like behaviors in rodents demonstrated that scopolamine attenuated the induced depression in mice partially by the regulation of the serotonin transporter, BDNF, and tryptophan hydroxylase 1 in the hippocampus and prefrontal cortex [102]. Supplementation with tryptophan, the precursor of serotonin synthesis, also ameliorated stress-induced depression-like behavior in mice by the improvement of neuroinflammation, mitochondrial energy metabolism, and increased expression of BDNF [103].

**Figure 1:**
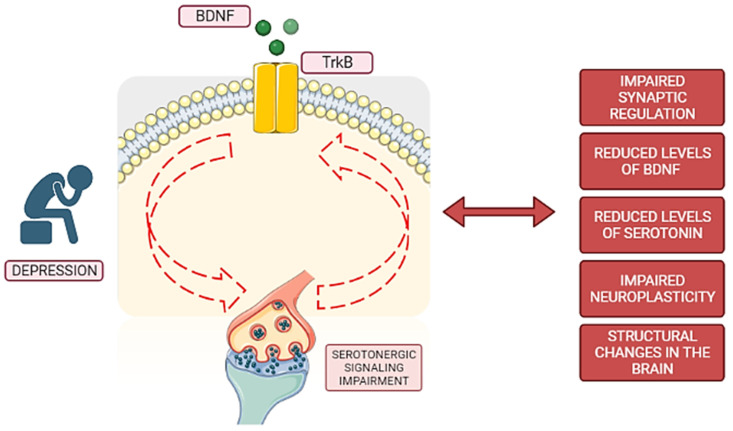
Simplified scheme of BDNF and serotonin interaction in depression. In depression, there exists a complex interplay between BDNF and serotonin. BDNF interacts with the TrkB receptor, thereby exerting its influence on serotonergic signaling pathways. Conversely, these pathways also impact BDNF signaling. Disruptions within these intricate pathways, indicated by red dashed arrows, are observed in major depressive disorder. Consequently, these disruptions contribute to structural changes in the brain, impaired neuroplasticity, compromised synaptic regulation, and reduced levels of both serotonin and BDNF. These cumulative effects ultimately promote or contribute to the development of depressive symptoms.

To summarize, serotonin and its receptors play critical roles in a variety of brain activities, and their dysregulation has been linked to several neuropsychiatric disorders. Serotonin and BDNF have a complex interaction because serotonergic pathways control BDNF expression, and BDNF, in turn, regulates the development and function of serotonergic neurons. Serotonin imbalances can influence BDNF levels, whereas BDNF promotes serotonergic neuron growth and plasticity. Serotonin and BDNF both contribute to synaptic regulation and structural changes in the brain, which are important in mood disorders such as depression. SSRIs increase BDNF gene expression, and serotonergic receptors further influence BDNF synthesis and signaling. Understanding the intricate relationship between serotonin and BDNF is crucial for advancing our knowledge of major depressive disorder, and recent studies have explored this connection, offering insights into potential therapeutic approaches for depression. Figure 1 represents a simplified summary of the BDNF and serotonin connection in major depression disorder.

## 5. Conclusions

In conclusion, BDNF is important to various physiological processes that include neuronal development, synaptic plasticity, neurogenesis, neuroprotection, and mood regulation. The connection between the BDNF signaling pathway and major depressive disorder is extensively studied and holds great importance in advancing our understanding and treatment of this alarming disorder. However, there is still much to uncover, as research in this field remains highly active. Despite the existence of effective treatments, relapse after treatment and resistance to standard treatment options are common, emphasizing the need for continuous research. Studies have found that reduced levels of BDNF are associated with an increased frequency of depressive symptoms, suggesting that BDNF plays an important role in the underlying mechanisms of this illness, besides other significant mechanisms, such as oxidative stress, imbalances in the HPA axis, and impaired neurotransmission.

The interplay of oxidative stress, HPA axis imbalance, serotonergic pathways, and BDNF plays a significant role in the development and progression of depression and other neuropsychiatric disorders. Exploring these interconnected mechanisms leads to advancements in the diagnosis and treatment of major depressive disorder, ultimately improving the lives of depressed individuals. Continued research in this field is crucial to uncovering new insights and developing more effective interventions for major depression disorder. The potential of BDNF-related treatments for various conditions including depression is promising, but there are some weaknesses that warrant consideration and further research, such as limited efficacy mainly due to delivery and central availability issues and safety concerns. Future directions may include a focus on personalized approaches to identify patient subgroups most likely to benefit from BDNF-related treatment, as well as studies to identify biomarkers and long-term efficacy and safety [104].

## Figures and Tables

**Table 2:** Summary of the BDNF and major depressive disorder connection.

Aspect	BDNF and Major Depressive Disorder Connection
Levels of BDNF	Reduced BDNF levels have been observed in individuals with major depressive disorder [45].
Changes in structure and function	Deficiencies or imbalances in BDNF levels may contribute to the development of depression by promoting structural and functioning changes [7], such as reduced dendritic complexity [37].
Serotonin influence	BDNF is influenced by serotonin, and serotonin activation can stimulate BDNF synthesis and release. Serotonin receptors can also modulate BDNF expression, influencing neuronal function and, consequently, mood regulation [59].
Neuroplasticity	BDNF is involved in neuroplasticity, which is crucial for synaptic connections and structural changes in the brain related to depressive disorder [37].
Antidepressant effects	Different antidepressants can enhance BDNF gene expression, contributing to their therapeutic effects [7].
Oxidative stress	Oxidative stress can lower BDNF production and damage its signaling pathways. The connection between oxidative stress and BDNF levels plays a significant role in the development and progression of depression [11].
Hypothalamic–pituitary–adrenal (HPA) axis dysregulation	Stress-induced HPA axis hyperactivity and the resulting increase in glucocorticoid levels diminish BDNF expression, playing an important role in the development of depression [10].

## Data Availability

Not applicable.
